# Antibiotic Use Prior to Hospital Presentation Among Individuals With Suspected Enteric Fever in Nepal, Bangladesh, and Pakistan

**DOI:** 10.1093/cid/ciaa1333

**Published:** 2020-12-01

**Authors:** Krista Vaidya, Kristen Aiemjoy, Farah N Qamar, Samir K Saha, Dipesh Tamrakar, Shiva R Naga, Shampa Saha, Caitlin Hemlock, Ashley T Longley, Kashmira Date, Isaac I Bogoch, Denise O Garrett, Stephen P Luby, Jason R Andrews

**Affiliations:** 1 Dhulikhel Hospital, Kathmandu University Hospital, Dhulikhel, Nepal; 2 Division of Infectious Diseases and Geographic Medicine, Stanford University School of Medicine, Stanford, California, USA; 3 Department of Pediatrics and Child Health, Aga Khan University Karachi, Pakistan; 4 Child Health Research Foundation, Department of Microbiology, Dhaka Shishu Hospital, Dhaka, Bangladesh; 5 Bangladesh Institute of Child Health, Dhaka Shishu Hospital, Sher-E-Bangla Nagar, Dhaka, Bangladesh; 6 Applied Epidemiology, Sabin Vaccine Institute, Washington, DC, USA; 7 National Foundation for the Centers for Disease Control and Prevention, Atlanta, Georgia, USA; 8 Global Immunization Division, Centers for Disease Control and Prevention, Atlanta, Georgia, USA; 9 Department of Medicine, University of Toronto, Toronto, Ontario, Canada

**Keywords:** enteric fever, typhoid, antimicrobial resistance, blood culture

## Abstract

**Background:**

Antibiotic use prior to seeking care at a hospital may reduce the sensitivity of blood culture for enteric fever, with implications for both clinical care and surveillance. The Surveillance for Enteric Fever in Asia Project (SEAP) is a prospective study of enteric fever incidence in Nepal, Bangladesh, and Pakistan. Nested within SEAP, we evaluated the accuracy of self-reported antibiotic use and investigated the association between antibiotic use and blood culture positivity.

**Methods:**

Between November 2016 and April 2019, we collected urine samples among a subset of SEAP participants to test for antibiotic use prior to the hospital visit using an antibacterial activity assay. All participants were asked about recent antibiotic use and had a blood culture performed. We used mixed-effect logit models to evaluate the effect of antimicrobial use on blood culture positivity, adjusted for markers of disease severity.

**Results:**

We enrolled 2939 patients with suspected enteric fever. Antibiotics were detected in 39% (1145/2939) of urine samples. The correlation between measured and reported antibiotic use was modest (κ = 0.72). After adjusting for disease severity, patients with antibiotics in their urine were slightly more likely to be blood culture positive for enteric fever; however, the effect was not statistically significant (prevalence ratio, 1.22 [95% confidence interval, .99–1.50]).

**Conclusions:**

The reliability of self-reported prior antibiotic use was modest among individuals presenting with fever to tertiary hospitals. While antibiotics are likely to reduce the sensitivity of blood culture, our findings indicate that there is still considerable value in performing blood culture for individuals reporting antibiotic use.

Enteric fever, caused by *Salmonella enterica* subspecies *enterica* serotypes Typhi and Paratyphi A, is a major cause of morbidity in South Asia [1]. Antibiotic treatment is one of the mainstays of enteric fever management and is responsible for a marked reduction in mortality from a disease that had a case fatality rate of 10%–25% in the pre–antibiotic era [2]. Unfortunately, accurate diagnosis of enteric fever remains a major challenge. Enteric fever is difficult to distinguish clinically from other causes of acute febrile illness [3–5], and there are no rapid diagnostic tests available to inform appropriate therapy with sufficient accuracy [6, 7]. Blood cultures have low sensitivity of 50%–60% and take 48–72 hours to provide results, which limits their utility in clinical decision-making [8].

Antibiotic use prior to seeking care at a hospital may reduce the sensitivity of blood culture for enteric fever [8, 9], with implications for both clinical care and surveillance. In resource-constrained environments, it is sometimes questioned whether there is sufficient value in performing blood culture among individuals who report recent antibiotic use, because of the perceived impact that antibiotic use has on the yield of blood cultures. However, ascertaining antimicrobial use accurately is difficult. In contexts where antimicrobials are easily available, few pharmaceutical records exist, and many people struggle to distinguish antibiotics from other medications such as antipyretics or antidiarrheal. Relying on self- or caregiver-reported antibiotic use can be misleading [10–13]. Urine antibacterial assays quantify antibiotic presence in the urine and are an appealing alternative to reported antibiotic use [14], particularly in settings where antibiotics are widely available and antibiotic knowledge and awareness are known to be low.

Easy access and indiscriminate use of antibiotics prior to formal care may be an important contributor to the emerging threat of antimicrobial resistance. Rising rates of antimicrobial resistance may undermine treatment effectiveness for enteric fever as well as other infectious diseases [15]. Critically, the empiric use of antibiotics in patients with nonspecific febrile illnesses, suspected of having enteric fever, may have implications for antibacterial resistance beyond that of typhoidal *Salmonella.* Therefore, measuring patterns of antibiotic use for febrile illness in enteric-fever endemic settings may enhance our understanding of the drivers of antimicrobial resistance.

To investigate patterns of antibiotic use among patients with acute febrile illness, we conducted a substudy among patients presenting to outpatient departments who were enrolled in the Surveillance for Enteric Fever in Asia Project (SEAP) study sites in Bangladesh, Nepal, and Pakistan. Our objectives were to characterize prehospital antibiotic use among patients with suspected enteric fever in South Asia according to demographic and clinical characteristics, evaluate the accuracy of self-reported antibiotic use, and investigate the relationship between antibiotic use and culture positivity.

## MATERIALS AND METHODS

### Overall Design

This study was nested within the SEAP study, a longitudinal surveillance study for typhoid and paratyphoid fevers in Bangladesh, Nepal, and Pakistan. SEAP study methods are presented elsewhere [16, 17]. Individuals presenting to outpatient departments with 3 or more consecutive days of fever in the prior 7 days were eligible to enroll in SEAP. We collected urine samples from a subset of this population to test for the presence of antimicrobial activity and inquired about their antibiotic use.

### Study Population

SEAP patients presenting to the outpatient department at any of the 6 study hospitals with 3 or more consecutive days of fever were eligible to participate. Participants were enrolled consecutively from September 2016 through April 2019 until the target sample size was reached. All study hospitals were tertiary facilities: 2 in Dhaka, Bangladesh; 1 in Kathmandu, Nepal; 1 in Kavrepalanchok, Nepal; and 2 in Karachi, Pakistan. While participants of any age were eligible to participate, the hospital study sites in Bangladesh serve primarily pediatric populations.

### Measurements and Definitions

The eligibility criteria of 3 or more consecutive days of fever in the previous 7 days was assessed by self-report or caregiver report. We interviewed all study participants, using a standardized questionnaire to ascertain demographic and clinical history. A sample of peripheral venous blood was collected from each participant, inoculated into a BACTEC Aerobic bottle or PED Plus bottle, and incubated in a BACTEC system for 5 days. One blood culture was performed per patient. Samples that indicated positivity in the BACTEC system were subcultured onto MacConkey agar plates and nonselective media (sheep blood agar). We identified the species with biochemical testing and confirmed serologically, using O and H antisera (BD laboratories), when available.

We also requested a sample of at least 3 mL of urine in a sterile collection cup from the participants before any antibiotics were prescribed or administered at the study facility. Urine was collected the same day blood was drawn for culture. These samples were either processed immediately or stored at 2°C–8°C and processed the following day or stored at −80°C and processed within a month. In an internal validation study of 10 samples, there was no difference in susceptibility between fresh samples and samples stored at −20°C and −80°C for up to 3 months’ storage time.

To test for the presence of antibiotics, we prepared a bacterial lawn of *Kocuria rhizophila* (previously *Micrococcus luteus*) (ATCC:9341) on Mueller-Hinton agar (Oxoid: CM033) with 5% sheep’s blood. *Kocuria rhizophila* has been previously used to detect low concentrations of antibiotics in biological fluids because of its high susceptibility to antibiotics [18, 19]. We placed a type of sterile blank disk (Oxoid: CT998) used in Kirby-Bauer disk diffusion testing on the lawn of bacteria and added 8–10 µL of the urine sample to the disk. After incubating the plate for 24 hours at 37°C, we measured the zone of inhibition around the disk. Any zone of inhibition around the disk was interpreted as detection of antibiotics in the urine. We did quality control of the media during the preparation of each batch, by testing the positive and negative control disks on the bacterial lawn.

We inquired about care-seeking and antibiotic use in the 7 days prior to presenting for care at the study facility by asking the following questions to all participants: “For this febrile illness, before arriving for this visit, did you/the participant seek any type of medical advice or treatment for your/their symptoms, such as a health clinic, other hospital, pharmacy, or private healthcare provider?” and “Before arriving for this visit, did you/the participant take any of the following medications, such as antibiotics, antipyretics, analgesics, and antidiarrheals?”

We did not collect additional information on the specific antibiotics consumed. We also collected information about household assets, education and self-reported clinical symptoms. If the participants were <15 years old, we asked these questions to their caregiver or guardian.

### Statistical Analysis

The primary outcome was the presence of antibiotics in the urine as measured by the biological assay described above. We reported the proportion of participants with antibiotics detected in their urine samples, along with 95% confidence intervals (CIs), stratified by country and age. We used principal component analysis to construct a wealth index variable from the household asset data for these analyses. We used binomial mixed-effect logit models for all the remaining analyses with random effects for hospital site and country to account for clustering. We report the results as prevalence ratios, calculated from the probability-logit transformations from the mixed models [20]. We calculated the sensitivity and specificity of reported antibiotic use with antibiotics detected in the urine as the reference standard.

We evaluated the relationship between antibiotics detected in the urine and blood culture positivity for *S.* Typhi/*S.* Paratyphi. We ran an unadjusted model (model 1) alongside a model adjusted for markers of disease severity (days unable to complete normal activity and hours spent in bed on the worst day of illness), days of fever, and age, which we hypothesized might confound the association between the antibiotic use and culture positivity.

We anticipated that 30% of the study patients would have antibiotics detected in their urine. To estimate prevalence across the 3 age groups with a width of the 95% CI of ±5% required 969 participants per site.

We used R version 3.6.0 software for all our analyses.

### Ethics Statement

All participants gave informed consent; in the case of minors, verbal assent and informed consent were provided by a parent or guardian. The study was approved by the Nepal Health Research Council, Bangladesh Institute of Child Health Ethical Review Committee, Pakistan Ethical Review Committee, the US Centers for Disease Control and Prevention, and the Institutional Review Board at Stanford University.

## RESULTS

We recruited 2939 individuals presenting to the outpatient department at SEAP study hospitals with a fever of 3 or more days in the past week. This included 999 participants in Bangladesh, 1001 in Nepal, and 939 in Pakistan. The median age of the participants was 10 years (interquartile range [IQR], 5–25 years), and 1246 (42.4%) of the participants were female. The median temperature at hospital presentation was 37.2 C (IQR, 36.7–38.2), and patients reported a median of 4 days (IQR, 3–6 days) of fever (Table 1). All participants had a blood culture, and 373 (12.7%) tested positive for *S.* Typhi or *S.* Paratyphi. Culture positivity was higher in Bangladesh (20.1%) and Pakistan (14.5%) and lower in Nepal (3.6%).

**Table 1.** Characteristics of Enrolled Patients With Enteric Fever by Country, Surveillance for Enteric Fever in Asia Project—Bangladesh, Nepal, and Pakistan, 2016–2019

Characteristic	Bangladesh (n = 999)	Nepal (n = 1001)	Pakistan (n = 939)	Total (N = 2939)
Female sex	423 (42.3)	413 (41.3)	410 (43.7)	1246 (42.4)
Age, y				
<2	77 (7.7)	19 (1.9)	32 (3.4)	128 (4.4)
2–4	370 (37.0)	105 (10.5)	102 (10.9)	577 (19.6)
5–15	552 (55.3)	232 (23.2)	310 (33.0)	1094 (37.2)
16–25	0 (0.0)	248 (24.8)	138 (14.7)	386 (13.1)
>25	0 (0.0)	397 (39.7)	357 (38.0)	754 (25.7)
Temperature at hospital presentation, °C				
Median	37.3	37.2	37.5	37.2
IQR	36.6–38	36.6–38.1	36.7–38.3	36.7–38.2
Days of fever before hospital presentation				
Median	4.0	4.0	5.0	4.0
IQR	3.0–6.0	3.0–5.0	3.0–6.0	3.0–6.0
Days unable to complete normal activity				
Median	2.0	3.0	3.0	3.0
IQR	0.0–4.0	2.0–5.0	2.0–5.0	1.0–5.0
Reported diarrhea	62 (6.2)	109 (10.9)	163 (17.4)	334 (11.4)
Diagnosed with enteric fever	816 (81.7)	198 (19.8)	466 (49.7)	1480 (50.4)
Blood culture positive for *S*. Typhi or *S.* Paratyphi	201 (20.1)	36 (3.6)	136 (14.5)	373 (12.7)
Antibiotics detected in urine	377 (37.7)	264 (26.4)	504 (53.8)	1145 (39.0)
Antibiotic use reported	412 (42.4)	417 (42.4)	430 (46.2)	1259 (43.7)
Prior care-seeking				
Hospital	97 (21.5)	171 (22.1)	340 (47.2)	608 (31.3)
Pharmacy	247 (56.7)	461 (59.6)	17 (2.4)	725 (37.7)
Clinic	3 (0.7)	144 (18.6)	239 (33.9)	386 (20.0)
Physician	78 (17.3)	5 (0.6)	210 (29.5)	293 (15.1)
Traditional healer	11 (2.4)	1 (0.1)	3 (0.4)	15 (0.8)
Wealth index quintile				
1 (lowest)	79 (18.0)	22 (5.2)	173 (34.5)	274 (20.1)
2	68 (15.5)	74 (17.4)	131 (26.1)	273 (20.0)
3	67 (15.3)	91 (21.4)	115 (22.9)	273 (20.0)
4	87 (19.8)	104 (24.5)	82 (16.3)	273 (20.0)
5 (highest)	138 (31.4)	134 (31.5)	1 (0.2)	273 (20.0)
Highest education, female head of household				
None	23 (5.2)	158 (39.7)	144 (27.6)	325 (23.8)
Primary	204 (45.8)	80 (20.1)	96 (18.4)	380 (27.9)
Secondary	130 (29.2)	93 (23.4)	133 (25.5)	356 (26.1)
Postsecondary	88 (19.8)	67 (16.8)	148 (28.4)	303 (22.2)

Data are presented as no. (%) unless otherwise indicated.

Abbreviation: IQR, interquartile range.

**Figure 1.**
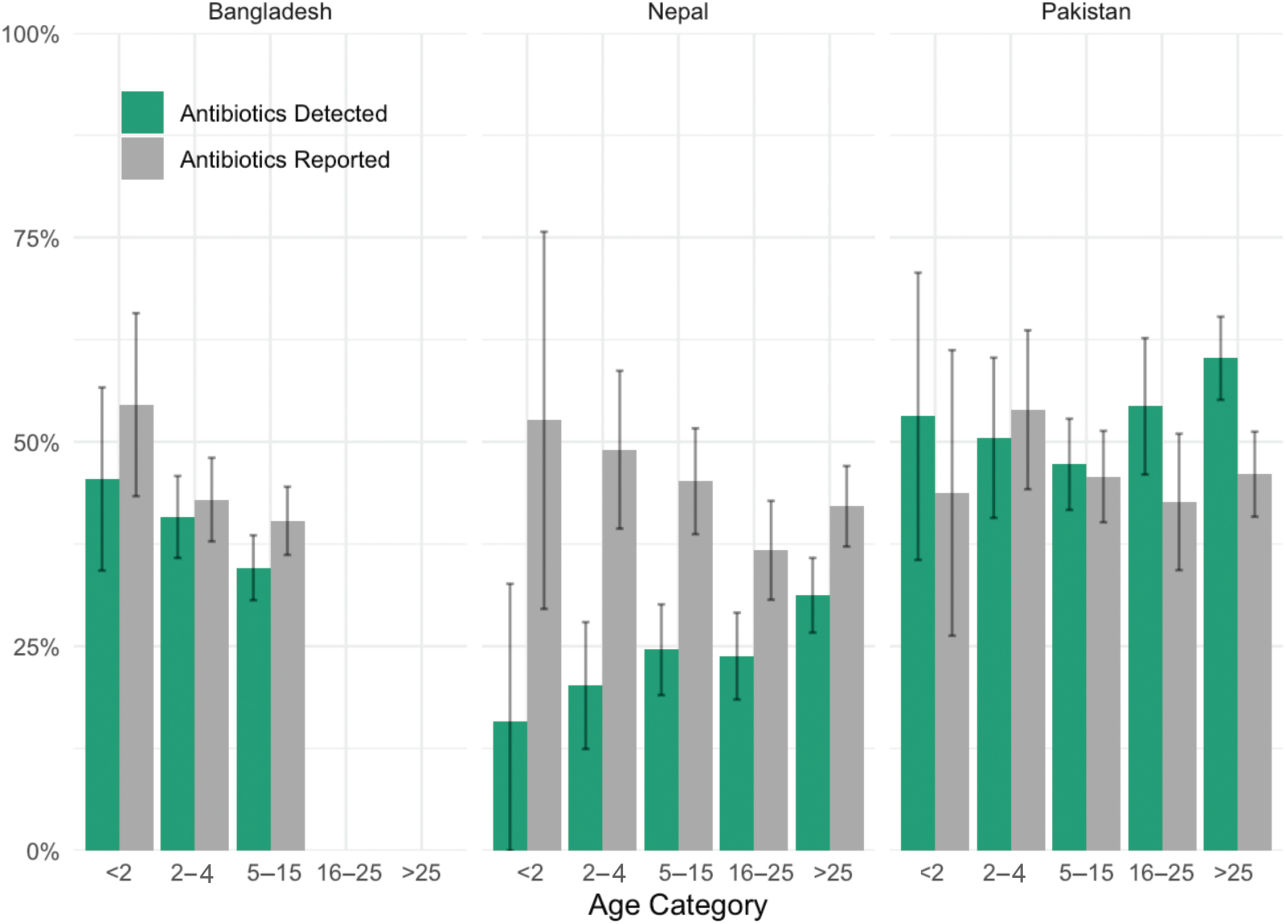
Differences between antibiotic detection and report across study country and age strata, Surveillance for Enteric Fever in Asia Project—Bangladesh, Nepal, and Pakistan, 2016–2019. Differences in the percentage of patients reporting antibiotic use and patients with antibiotics detected in the urine by country and age category among 2939 outpatients with ≥3 consecutive days of fever in Bangladesh, Nepal, and Pakistan. Vertical line in the middle of each bar depicts the 95% confidence interval around the percentage.

Overall, we detected antibiotics in the urine samples of 1145 (39%) participants: 377 (37.7%) in Bangladesh, 264 (26.4%) in Nepal, and 504 (53.8%) in Pakistan. In contrast, 1259 (43.7%) participants reported taking antibiotics prior to seeking care: 412 (42.4%) in Bangladesh, 417 (42.4%) in Nepal, and 430 (46.2%) in Pakistan (Table 1). In Bangladesh and Nepal, reported antibiotic use was higher than the detected antibiotics across all age groups (Figure 1).

**Figure 2.**
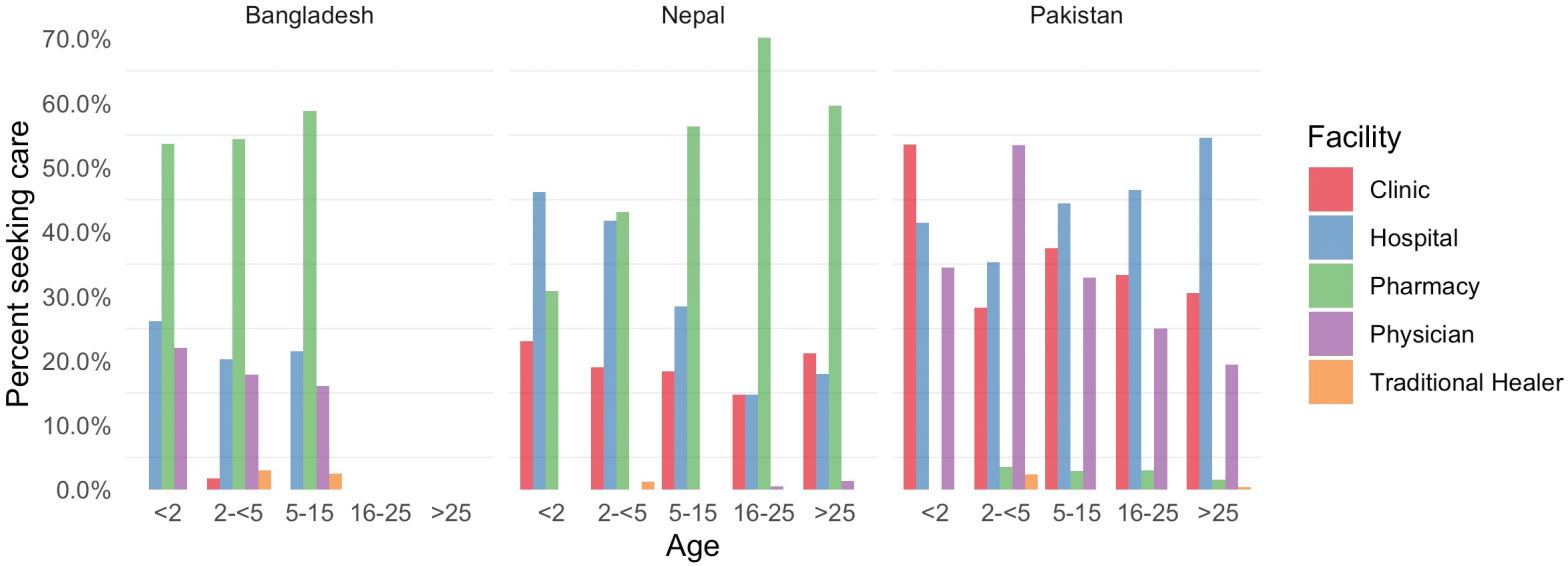
Age and facility care-seeking for current illness by country, Surveillance for Enteric Fever in Asia Project—Bangladesh, Nepal, and Pakistan, 2016–2019. Percentage of patients seeking care at a clinic, hospital, pharmacy, physician, or traditional healer prior to presenting to the study site among 2939 outpatients with ≥3 consecutive days of fever in Bangladesh, Nepal, and Pakistan.

When asked about care-seeking for the current illness prior to presentation at the study hospital, the most common location reported was a pharmacy, followed by a hospital or clinic. However, there were substantial differences between countries. For example, only 2.4% of the participants sought care at a pharmacy in Pakistan compared with 56.7% in Bangladesh and 59.6% in Nepal (Table 1). In Nepal, the percentage of children taken to the hospital decreased with age, while the percentage of patients visiting a pharmacy increased with age (Figure 2). However, this finding was not observed in other sites.

Antibiotic detection in urine was positively associated with having ≥7 days of fever (prevalence ratio [PR], 1.32 [95% CI, 1.19–1.45]), temperature of 38.3 C at presentation (PR, 1.19 [95% CI, 1.06–1.32]), having ≥3 days of being unable to conduct normal activities (PR, 1.14 [95% CI, 1.03–1.26]), and having diarrhea (PR, 1.19 [95% CI, 1.03–1.35]) (Table 2). Seeking care at a hospital, clinic, or pharmacy or with a physician for the current illness prior to presenting to the study hospital was also associated with a higher probability of detecting antibiotics in the urine samples (Table 2). There was no apparent association between wealth or education and antibiotics being detected in the urine (Table 2). These associations were consistent at the country level as well, barring the association between diarrhea and antibiotic detection in Bangladesh and seeking care at a pharmacy and antibiotic detection in Nepal (Table 3).

**Table 2.** Antibiotic Detection in Urine by Demographic and Behavioral Factors, Surveillance for Enteric Fever in Asia Project—Bangladesh, Nepal, and Pakistan, 2016–2018

Characteristic	Antibiotics Detected (n = 1145), No. (%)	Total (N = 2939), No.	PR (95% CI)	*P* Value
Age, y				
<2	55 (43.0)	128	Ref	
2–4	223 (38.8)	575	0.91 (.70–1.14)	.429
5–15	394 (36.0)	1093	0.83 (.64–1.05)	.122
16–25	134 (34.7)	386	0.96 (.72–1.22)	.753
>25	339 (45.0)	754	1.11 (.88–1.35)	.363
Sex				
Male	652 (38.6)	1691	Ref	
Female	493 (39.6)	1245	1.02 (.92–1.12)	.694
Fever duration ≥7 d				
No	859 (36.5)	2356	Ref	
Yes	279 (50.0)	558	1.32 (1.19–1.45)	<.001
Temperature at presentation 38.3 C				
No	817 (39.6)	2064	Ref	
Yes	236 (45.0)	524	1.19 (1.06–1.32)	.004
≥3 d unable to conduct activity				
No	399 (35.7)	1118	Ref	
Yes	691 (42.3)	1635	1.14 (1.03–1.26)	.012
Reported diarrhea				
No	983 (37.8)	2600	Ref	
Yes	162 (48.5)	334	1.19 (1.03–1.35)	.018
Prior to enrollment, patient sought care at a hospital				
No	786 (34.1)	2302	Ref	
Yes	340 (56.1)	606	1.54 (1.40–1.69)	<.001
Prior to enrollment, patient sought care at a pharmacy^a^				
No	862 (39.9)	2160	Ref	
Yes	246 (33.9)	725	1.20 (1.07–1.33)	.003
Prior to enrollment, patient sought care at a clinic				
No	922 (36.8)	2508	Ref	
Yes	201 (52.2)	385	1.31 (1.15–1.47)	<.001
Prior to enrollment, patient sought care with a physician				
No	950 (36.4)	2608	Ref	
Yes	171 (58.6)	292	1.43 (1.24–1.62)	<.001
Prior to enrollment, patient sought care with a traditional healer				
No	1119 (38.7)	2893	Ref	
Yes	7 (46.7)	15	1.10 (.54–1.75)	.761
Wealth index quintile				
1 (lowest)	125 (45.8)	273	Ref	
2	126 (46.2)	273	1.10 (.90–1.30)	.333
3	101 (37.1)	272	0.92 (.71–1.15)	.511
4	104 (38.1)	273	1.05 (.82–1.27)	.697
5 (highest)	86 (31.5)	273	1.03 (.80–1.26)	.803
Female head of household, highest education attained				
None	123 (38.0)	324	Ref	
Primary	161 (42.5)	379	1.14 (.92–1.36)	.224
Secondary	134 (37.6)	356	0.95 (.75–1.17)	.648
Postsecondary	128 (42.2)	303	1.02 (.80–1.26)	.848

PRs are estimates using mixed-effect models with random effects for country and site hospital and are adjusted for age.

Abbreviations: CI, confidence interval; PR, prevalence ratio.

^a^Because of heterogeneity in care seeking and antibiotic use by country, antibiotic use among individuals who sought care at a pharmacy appears lower than those who did not seek care at a pharmacy; however, when accounting for differences by country in the mixed-effect model, the PR for this association is higher.

**Table 3.** Country-level Associations With Antibiotic Detection in Urine, Surveillance for Enteric Fever in Asia Project—Bangladesh, Nepal, and Pakistan, 2016–2019

Characteristic	Bangladesh		Nepal		Pakistan	
	PR (95% CI)	*P* Value	PR (95% CI)	*P* Value	PR (95% CI)	*P* Value
Age, y						
<2	Ref		Ref		Ref	
2–4	0.88 (.64–1.16)	.398	1.12 (.34–2.84)	.842	0.95 (.59–1.31)	.795
5–15	0.75 (.53–1.01)	.061	1.41 (.47–3.20)	.518	0.89 (.57–1.22)	.527
16–25	…		1.47 (.49–3.28)	.463	1.02 (.67–1.36)	.900
>25	…		1.83 (.65–3.72)	.230	1.13 (.80–1.43)	.434
Female sex	1.16 (.98–1.34)	.078	0.92 (.73–1.13)	.421	0.98 (.86–1.09)	.711
Fever duration ≥7 d^a^	1.52 (1.29–1.75)	<.001	1.17 (.88–1.50)	.262	1.22 (1.06–1.36)	.006
Temperature at presentation >38.3 C^a^	1.34 (1.11–1.57)	.004	1.39 (1.04–1.78)	.027	1.02 (.87–1.16)	.810
≥3 d unable to conduct activity^a^	1.13 (.95–1.32)	.172	1.24 (.98–1.54)	.067	1.15 (1.01–1.29)	.042
Diarrhea^a^	0.92 (.63–1.26)	.631	1.19 (.86–1.58)	.275	1.25 (1.09–1.40)	.003
Prior care-seeking for current illness^a^						
Hospital	1.65 (1.34–1.94)	<.001	2.03 (1.65–2.42)	<.001	1.25 (1.11–1.37)	<.001
Pharmacy	1.64 (1.39–1.88)	<.001	0.89 (.70–1.11)	.292	1.05 (.61–1.44)	.843
Clinic	2.11 (.65–2.63)	.146	1.45 (1.11–1.83)	.007	1.19 (1.05–1.32)	.008
Physician	2.11 (1.77–2.39)	<.001	1.29 (.29–2.90)	.698	1.06 (.91–1.20)	.454
Traditional healer	1.36 (.63–2.08)	.377	0.00 (.00–3.81)	.911	0.57 (.06–1.56)	.441
Wealth index quintile						
1 (lowest)	Ref		Ref		Ref	
2	0.93 (.59–1.34)	.744	1.19 (.52–2.13)	.647	1.15 (.92–1.36)	.199
3	0.75 (.44–1.15)	.210	0.81 (.33–1.66)	.616	1.05 (.82–1.27)	.683
4	1.04 (.68–1.43)	.849	0.71 (.28–1.49)	.400	1.23 (.97–1.46)	.083
5 (highest)	0.99 (.67–1.34)	.940	0.88 (.37–1.70)	.731	–	–
Female head of household, highest education attained						
None	Ref		Ref		Ref	
Primary	1.07 (.61–1.55)	.796	0.68 (.39–1.12)	.139	1.23 (.96–1.47)	.092
Secondary	0.76 (.38–1.27)	.351	0.73 (.44–1.15)	.192	1.17 (.92–1.40)	.177
Postsecondary	0.87 (.44–1.41)	.638	0.80 (.46–1.30)	.395	1.19 (.95–1.41)	.117

Abbreviations: CI, confidence interval; NaN, xxx; PR, prevalence ratio.

^a^Adjusted for age.

Individuals who had antibiotics detected in their urine were more likely to be blood culture positive for *S*. Typhi or *S.* Paratyphi A compared with individuals who had no antibiotics in their urine (PR, 1.33 [95% CI, 1.10–1.60]). When adjusting for the duration of fever, days in bed, diarrhea, age, and wealth, the prevalence ratio decreased to 1.22 (95% CI, .99–1.50) and was no longer statistically significant (*P* = .065) (Table 4). When restricting to patients with a clinical diagnosis of enteric fever, individuals with antibiotics in their urine were still more likely to be blood culture positive for *S*. Typhi or *S.* Paratyphi compared with those who had no antibiotics detected in their urine in the unadjusted analysis (PR, 1.29 [95% CI, 1.05–1.55]; *P* = .014).

**Table 4.** Antibiotic Detection in the Urine and Blood Culture Positivity, Surveillance for Enteric Fever in Asia Project—Bangladesh, Nepal, and Pakistan, 2016–2019

Detection	Model 1 (Unadjusted)		Model 2^a^		Model 3^b^	
	PR (95% CI)	*P* Value	PR (95% CI)	*P* Value	PR (95% CI)	*P* Value
Overall	1.33 (1.1–1.6)	.004	1.22 (.99–1.5)	.065	1.29 (1.05–1.55)	.014
Country						
Bangladesh	1.34 (1.05–1.67)	.019	1.25 (.96–1.6)	.102	1.28 (1.01–1.6)	.041
Nepal	1.77 (.92–3.3)	.088	1.38 (.52–3.54)	.518	0.69 (.19–2.27)	.557
Pakistan	1.19 (.87–1.59)	.277	1.26 (.9–1.71)	.171	1.38 (.97–1.89)	.074

Abbreviations: CI, confidence interval; PR, prevalence ratio.

^a^Model 2: Adjusted for fever duration, days unable to complete normal activity, temperature at presentation, age, wealth.

^b^Model 3: Restricted to participants with a clinically suspected enteric fever diagnosis.

The sensitivity and specificity of reported antibiotic use compared to the detection of antibiotics in the urine were 70.2% (95% CI, 57.9%–80.2%) and 72.6% (95% CI, 66.1%–79.1%), respectively. Correlation between measured and reported antibiotic use was modest in all 3 countries but was higher in Bangladesh (κ = 0.74) and Nepal (κ = 0.73) than in Pakistan (κ = 0.62). The sensitivity of reported antibiotic use was slightly higher for parents and guardians compared to self-report (Table 5).

**Table 5.** Sensitivity and Specificity of Antibiotic Use Reporting by Country and Caregiver Type, Surveillance for Enteric Fever in Asia Project—Bangladesh, Nepal, and Pakistan, 2016–2019

Variable	TP^a^	FN^b^	TN^c^	FP^d^	Sensitivity, % (95% CI)	Specificity, % (95% CI)
Overall	781	342	1280	478	70.2 (57.9–80.2)	72.6 (66.1–79.1)
Country						
Bangladesh	288	74	485	124	75.2 (59.2–86.4)	80.5 (74.2–86.7)
Nepal	204	56	509	213	78.9 (64.5–88.5)	69.2 (61.3–77.0)
Pakistan	289	212	286	141	56.6 (40.0–71.8)	67.1 (58.5–75.6)
Respondent type^e^						
Self	279	152	435	161	67.4 (51.9–79.8)	69.5 (59.5–79.5)
Parent or guardian	454	166	770	279	72.3 (58.6–82.8)	74.8 (67.0–82.7)
Other relative	47	24	71	38	70.3 (52.0–83.7)	68.6 (56.7–80.5)
Age, y						
<2	35	20	42	31	62.3 (42.0–79.0)	51.8 (36.3–67.2)
2–4	161	56	248	100	74.9 (60.8–85.2)	67.7 (57.5–77.8)
5–15	284	101	507	174	75.8 (62.9–85.2)	72.4 (63.8–80.9)
16–25	85	46	187	63	65.6 (49.0–79.0)	77.8 (69.2–86.5)
>25	216	119	296	110	62.7 (47.5–75.8)	77.7 (69.7–85.7)

Abbreviations: CI, confidence interval; FN, false negative; FP, false positive; TN, true negative; TP, true positive.

^a^True positive: Number of patients who reported antibiotic use and had antibiotics detected in their urine.

^b^False negative: Number of patients who did not report antibiotic use who did have antibiotics detected in their urine.

^c^True negative: Number of patients not reporting antibiotic use who did not have antibiotics detected in their urine.

^d^False positive: Number of patients who reported antibiotic use who did not have antibiotics detected in their urine.

^e^Adjusted for age.

## DISCUSSION

Antibiotic use prior to presentation to hospitals may have important implications for diagnostic evaluation by clinicians and for pathogen surveillance systems. However, the reliability of self-report of recent antibiotic use is unclear, and whether individuals who report prior antibiotic use should be included in blood culture–based surveillance systems has been debated. To address these knowledge gaps, we evaluated antibiotic use, self-report, and blood culture positivity among a subset of outpatients presenting to hospitals in Bangladesh, Nepal, and Pakistan. Antibiotic use across all 3 countries was high, with 1 of every 3 participants having antibiotic activity confirmed in their urine assay upon presenting at the tertiary care study site. Patients presenting with more severe disease (higher temperature at presentation, more days of fever, diarrhea, and longer time unable to complete normal activities) were more likely to have taken antibiotics than those with less severe disease. Many patients had sought care at other facilities prior to presenting at the study-site facility; of these, those who sought care at other hospitals or with physicians were more likely to have taken antibiotics. In contrast to what is often presumed, patients who had taken antibiotics prior to presentation were more likely to be culture positive than those who did not have evidence of antibiotics in their urine. Finally, the accuracy of self-reported or caregiver-reported antibiotic use compared to antibiotics detected in the urine was modest in all 3 countries.

The impact of antimicrobial use on the sensitivity of blood culture of *S.* Typhi and *S*. Paratyphi is not well characterized [21]. A recent meta-analysis found that reported prior antibiotic use was associated with a 34% decreased sensitivity of blood culture, although the size of the studies was small and there was substantial heterogeneity between them. Additionally, all studies were performed between the 1970s and 1990s, when different antibiotics were used for treatment of suspected enteric fever than those commonly used today. To investigate the impact of currently used antibiotics (eg, azithromycin, cefixime, and ceftriaxone) on culture sensitivity, a reference standard not strongly affected by antibiotics is needed. Historically, studies have used bone marrow cultures, which are less rapidly affected by antibiotics due to higher bacterial concentrations; however, this procedure is invasive, painful, and not widely performed today. Newer serologic diagnostics have demonstrated promise for improving the diagnosis of typhoid and may be useful as non-culture-based references for future studies [22, 23].

While we found higher culture positivity among individuals with prior antibiotic use, we believe that this was due to confounding whereby individuals who continued to have fever despite continued antibiotic use are more likely to have typhoid than other illnesses. The duration of fever in typhoid is characteristically longer than the other common causes of fever. Even after antibiotic use, the average fever clearance time in clinical trials for enteric fever treatment is around 5 days [24]. Persistent fever despite antibiotic use may therefore be a marker of enteric fever. This may be particularly true if individuals received antibiotics to which their *S.* Typhi or *S.* Paratyphi A isolate was not susceptible. Because antimicrobial resistance was widespread and fairly homogeneous within study sites, and the assay we used could not distinguish between specific antibiotics, we were unable to assess whether antimicrobial resistance mediated the effects of prior antibiotic use on culture positivity.

While self- or caregiver-reported antibiotic use is widely thought to be inaccurate, few studies have documented the validity of reported antibiotic use against a urine antibacterial assay in the South Asia region [10–13]. Our results were comparable to a study of young children with respiratory tract infections in the Philippines, where the sensitivity and specificity of caregiver-reported antibiotic use were 59% and 71%, respectively [25]. The low sensitivity documented in our study indicates that both clinicians and researchers should use caution when interpreting self- and caregiver-reported antibiotic use.

The results of this study should be interpreted within the context of the limitations of the design and study procedures. Biologic assays for antibiotic metabolites in urine may have limited sensitivity, which could lead to underestimation of prior antibiotic use and bias associations between demographic and clinical features, likely toward the null [26]. Their accuracy likely varies by antibiotic class, the number of doses taken, and recentness of the last dose. We did not have data about timing of doses or concentrations of antibiotics or their metabolites in urine over time. The assay used in our study did not enable us to distinguish between different antibiotic classes. It is possible that nonantibiotic metabolites in the urine or other chemical properties (eg, pH, urea, and ammonia concentrations) could have inhibited *Kocuria* growth, leading to false-positive results [27–29]. Although we did not observe bacterial inhibition in urine collected from healthy controls individuals during a pilot phase, it is possible that these chemical properties are altered in individuals with acute illness.

Despite these limitations, this is the largest study to date evaluating prior antibiotic use among enteric fever suspects and the associations with culture positivity and self-reported use. We unexpectedly found that individuals with reported antibiotic use were more likely to be culture positive for enteric fever; however, this relationship was confounded by disease severity. While antibiotics may reduce the sensitivity of blood culture, our findings indicate that there is still considerable value in performing blood culture among individuals reporting antibiotic use. Given the continuing emergence and spread of antimicrobial resistance among typhoidal *Salmonella* [15, 30], a better understanding of the relationship between antibiotic usage patterns and typhoid detection and resistance may improve our ability to monitor and respond to this epidemic.

## Notes

***Financial support.*** This work was supported by the Bill & Melinda Gates Foundation (BMGF) (investment ID OPP1113007). Additionally, there is an agreement (subcontract/subgrant) between the Sabin Vaccine Institute (primary grantee for the Surveillance for Enteric Fever in Asia Project grant from BMGF) and the CDC Foundation.

***Supplement sponsorship.*** This supplement is sponsored by the Sabin Vaccine Institute and made possible by a grant from the Bill & Melinda Gates Foundation.

***Potential conflicts of interest.*** S. S. has received grants from the World Health Organization, BMGF, GlaxoSmithKline, Pfizer, Sanofi Pasteur, and Edinburgh University, outside the submitted work. D. T. and J. R. A. received grants from BMGF, during the conduct of the study. All other authors report no potential conflicts of interest. All authors have submitted the ICMJE Form for Disclosure of Potential Conflicts of Interest. Conflicts that the editors consider relevant to the content of the manuscript have been disclosed.
